# Sedative Efficacy of Propofol in Patients Intubated/Ventilated after Coronary Artery Bypass Graft Surgery

**DOI:** 10.5812/aapm.17109

**Published:** 2014-02-28

**Authors:** Nahid Aghdaii, Frouzan Yazdanian, Seyedeh Zahra Faritus

**Affiliations:** 1 Rajaei Cardiovascular, Medical and Research Center, Iran University of Medical Sciences, Tehran, Iran

**Keywords:** Propofol, Analgesics, Coronary Artery Bypass, Deep Sedation, Midazolam, Airway Extubation, Length of Stay

## Abstract

**Background:**

Sedation after open heart surgery is important in preventing stress on the heart. The unique sedative features of propofol prompted us to evaluate its potential clinical role in the sedation of post-CABG patients.

**Objectives:**

To compare propofol-based sedation to midazolam-based sedation after coronary artery bypass graft (CABG) surgery in the intensive care unit (ICU).

**Patients and Methods:**

Fifty patients who were admitted to the ICU after CABG surgery was randomized into two groups to receive sedation with either midazolam or propofol infusions; and additional analgesia was administered if required. Inclusion criteria were as follows: patients 40-60 years old, hemodynamic stability, ejection fraction (EF) more than 40%; exclusion criteria included patients who required intra-aortic balloon pump or inotropic drugs post-bypass. The same protocol of anesthetic medications was used in both groups. Depth of sedation was monitored using the Ramsay sedation score (RSS). Invasive mean arterial pressure (MAP) and heart rate (HR), arterial blood gas (ABG) and ventilatory parameters were monitored continuously after the start of study drug and until the patients were extubated.

**Results:**

The depth of sedation was almost the same in the two groups (RSS=4.5 in midazolam group vs 4.7 in propofol group; P = 0.259) but the total dose of fentanyl in the midazolam group was significantly more than the propofol group (12.5 mg/hr vs 4 mg/hr) (P = 0.0039). No significant differences were found in MAP (P = 0.51) and HR (P = 0.41) between the groups. The mean extubation time in patients sedated with propofol was shorter than those sedated with midazolam (102 ± 27 min vs 245 ± 42 min, respectively; P < 0.05) but the ICU discharge time was not shorter (47.5 hr vs 36.3 hr, respectively; P = 0.24).

**Conclusions:**

Propofol provided a safe and acceptable sedation for post-CABG surgical patients, significantly reduced the requirement for analgesics, and allowed for more rapid tracheal extubation than midazolam but did not result in earlier ICU discharge.

## 1. Background

Sedation after open heart surgery is very important because most cardiac surgeons and anesthetists prefer CABG patients to awake slowly, to prevent any stress on the heart. Most patients require both sedation and analgesia to promote natural sleep, facilitate assisted ventilation, and modulate physiologic responses to stress (e.g. tachycardia and hypertension) (1-3). Pain after the cardiac surgery can have many sources, including the sternotomy incision, chest tubes, and leg incisions. Some of the deleterious effects of postoperative pain after cardiac surgery are due to the stress response and enhanced sympathetic tone (4, 5), which can increase the heart rate, pulmonary vascular resistance, myocardial work, and myocardial oxygen consumption. Post-cardiac surgical pain can also negatively affect the respiratory system. Inadequate sedative techniques may adversely affect morbidity and even mortality rates in the intensive care unit (ICU) (6-9). Currently, several sedatives, analgesics, and other agents are used to achieve these goals alone, or in combination (6, 10-12); however, these drugs may cause other problems. Propofol and midazolam are commonly used for the care of post–coronary artery bypass graft (CABG) patients in the ICU, but compared with midazolam, propofol provides equal or better control in maintaining sedation (6, 13, 14) and more rapid recovery (15-17), more rapid extubation when the sedation is terminated (18, 19) and less requirement for analgesic drugs to control pain (15-17, 20). In some studies, rapid extubation has equated to a shorter ICU stay (3, 21, 22); however, in other studies, the duration of stay was the same (23, 24). The unique sedative characteristics of propofol prompted us to evaluate its potential clinical role in the sedation of post cardiac surgery patients and to compare a propofol-based sedation regimen to a midazolam-based sedation that is currently used for the care of post–coronary artery bypass graft (CABG) patients in the ICU.

## 2. Objectives

To evaluate the potential clinical role of propofol in the sedation of post cardiac surgery patients and to compare the sedative properties, safety profiles, cardiovascular responses and ventilation and extubation characteristics of propofol with those of the commonly used i.v. sedative agent midazolam in the cardiac intensive care unit.

## 3. Patients and Methods

In this randomized clinical trial, all the consecutive patients undergoing elective CABG in Shahid Rajaei Hospital, Tehran, Iran were enrolled in the study. After the study protocol was approved by the Ethics Committee of Shahid Rajaei Hospital, all of the 50 adult patients signed informed written consent to participate in this study. Fifty adult patients, who had undergone coronary artery bypass graft surgery, were postoperatively assigned to one of two treatment regimens for sedation, if it was expected that they would require a minimum of 8 h mechanical ventilation after the surgery. These patients were ASA physical status II, 40-60 years old and had ejection fraction (EF) ≥ 40% who were scheduled for elective coronary artery bypass graft surgery under general anesthesia. Exclusion criteria were patients with underlying and co-existing diseases (hypertension, diabetes mellitus, renal disease, endocrine diseases), opium addiction and patients requiring post-bypass intra-aortic balloon pump or inotropic drugs. Protocol of anesthetic medications used in all of patients was the same and according to standard clinical practices. Before the induction of anesthesia, all patients were pre-medicated with 1 mg intramuscular lorazepam and 0.1 mg/kg morphine sulfate one hour before entering the operating room. Induction of anesthesia was performed under the monitoring of ECG, pulse oximetry and invasive arterial blood pressure with 0.2 mg/kg etomidate, 2.5 μg/kg sufentanil and 0.2 mg/kg cisatracurium and maintenance of anesthesia after the insertion of central venous line was achieved with continuous infusion of midazolam, sufentanil, and atracurium in both groups. 

### 3.1. Interventions

On arrival in the ICU, patients were allocated randomly, using sealed envelopes provided by the supervisor of ICU to receive i.v. infusions of either midazolam (Midazolex, Manufactured by: EX IR Iran) or propofol (Propofol 1% Fresenius Vial 50 cc, Manufactured by: Fresenius Kabi Austria) while being mechanically ventilated, together with the short‐acting opioid fentanyl by continuous infusion, for analgesia if required. An initial loading dose infusion of midazolam or propofol was given to rapidly achieve a steady‐state plasma concentration. The loading dose infusion of midazolam was 0.05 mg/kg over 10 min followed by a maintenance infusion of 0.04- 0.1 mg/kg/h using a peripheral or central vein. Propofol was given undiluted (ropofol 1%) as an infusion of 1-3 mg/kg/hr, after a loading dose infusion of up to 1 mg/kg over 10 min. Fentanyl was infused at 1-5 µg/kg/hr after a bolus of 1 µg/kg if the patient was in pain. 

### 3.2. Study Measures

**Figure 1.**
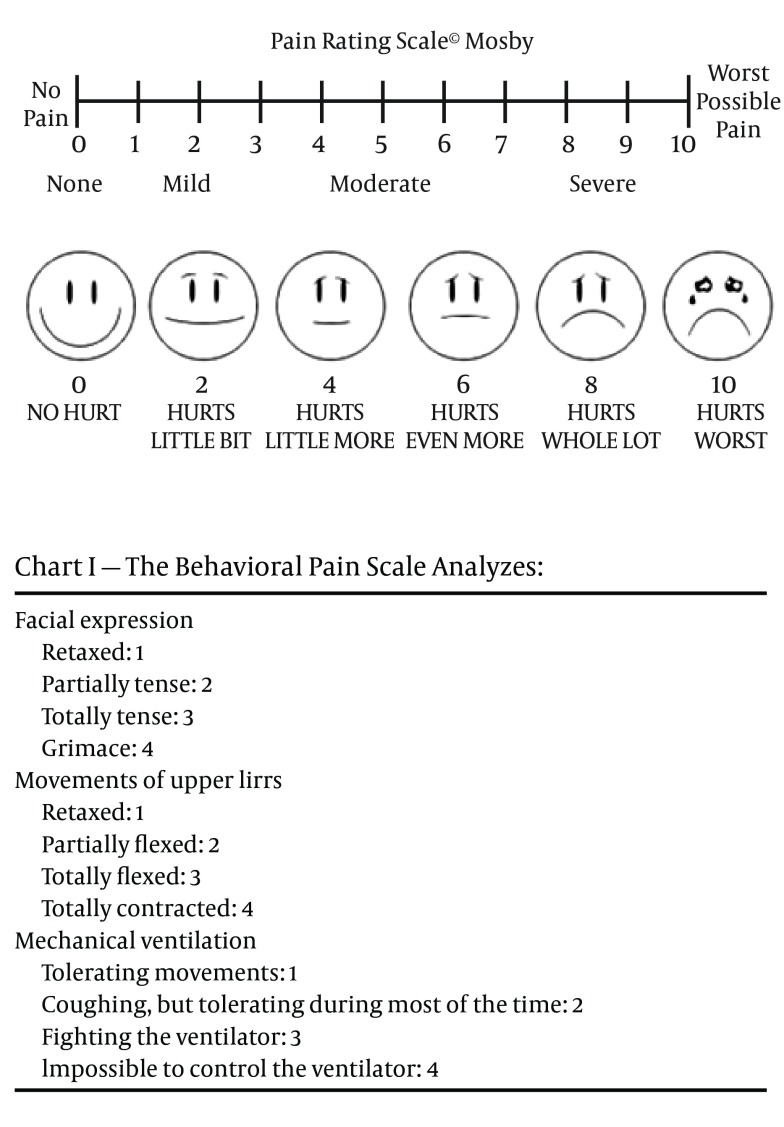
The Behavioral Pain Scale Analysis

Since the most common sedation scale in the previous studies was the Ramsay scale, the degree of sedation was measured and recorded hourly using the Ramsay Sedation Score (RSS) (Box 1) and patients were maintained at RSS= 3-5 by adjusting the sedative regimen. Pain measurement was done by the Behavioral Pain Scale (Figure 1), which is validated for use in the mechanically ventilated patients. Also, the staff determined the need for analgesia by direct communication with the patient or by monitoring the signs of pain (e.g. sweating, increased blood pressure, and elevated heart rate above 20% or more of the patient's baseline blood pressure and heart rate) and checking again for pain. Patients were ventilated mechanically in SIMV mode with oxygen under pulse oxymetry and arterial blood gas monitoring. Guidelines used for the weaning process included a decrease in the FIO_2_ every 30 minutes by 0.1, while a SpO_2_ ≥ 95% was maintained until an FIO_2_ ≤ 0.4 was reached. Extubation time was defined as the time from cessation of sedative infusion to extubation. The sedative and analgesic infusion was discontinued, in preparation for the extubation, when there was no evidence of bleeding and the patient was awake, cooperative and comfortable, cardiovascularly stable, normothermic, and with an acceptable blood gas on FIO_2_ ≤ 0.4, positive end-expiratory pressure ≤ 5 cm H2O, pressure support ≤ 10 cm H_2_O, tidal volume ≥ 5 mL/kg, and spontaneous respiratory rate < 20/min. The patient status was assessed every 30 minutes and recorded every 2 hours. Blood pressure and heart rate were monitored continuously but recorded every 30 minutes during the first hour after the start of study drug and hourly until study drug was stopped. SpO_2_ was monitored continuously. ABG, Na, K, Cl, Ca, Hgb, Hct, and lactate were checked every two hours until the extubation. Venous samples were taken for routine hematological (hemoglobin, hematocrit, coagulation panel) and biochemical (electrolytes, urea, creatinine, glucose, phosphate and calcium) profiles immediately on arrival in the ICU, and then at 24 and 48 h; but the results are not shown in this report. Discharge from the ICU was performed if there were no signs of neurological (Ramsay sedation score 2), respiratory (SpO_2_ ≥ 92%, p_a_O_2 _> 69 mmHg, p_a_CO_2_ = 35 to 45 mmHg, inspired O_2 _< 3 l/minute), hemodynamic (no catecholamines or inotropes, no significant fluid deficit) or surgical (no anticipated surgical complication) impairment and if the pain score on the pain rating scale or visual analogue scale (VAS) was < 4.

### 3.3. Statistical Analysis

The statistical analysis of data was performed using the SPSS Version 15.0 statistical software (SPSS Inc, Chicago, IL) The intragroup differences of the circulatory variables recorded over the time were analyzed using the repeated measures analysis of variance. Differences in the mean values of circulatory variables and analgesic requirement were analyzed using independent samples t test. The categorical variables in the two groups were analyzed using Chi-square test or Fisher's exact test. Extubation time and ICU stay time variables were analyzed by K-S test for assessing the normal distribution; and if not distribute normally, Mann-Withney U test was used for the analysis. The hypothesis of this study was that there would be a clinically meaningful difference in the hemodynamic responses, extubation time, ICU stay and analgesic requirement between the groups receiving two different sedative drugs. The quantitative data were expressed as mean ± SD. A P value of ≤ 0.05 was considered statistically significant for all tests.

**Box 1.** Ramsay Sedation Scale

	Ramsay Sedation Scale
**1**	Anxious and agitated, or restless, or both
**2**	Cooperative, oriented, and tranquil
**3**	Responding to commands only
**4**	Brisk response to glabellar tap
**5**	Sluggish response to glabellar tap
**6**	No response to light glabellar tap

## 4. Results

**Figure 2.**
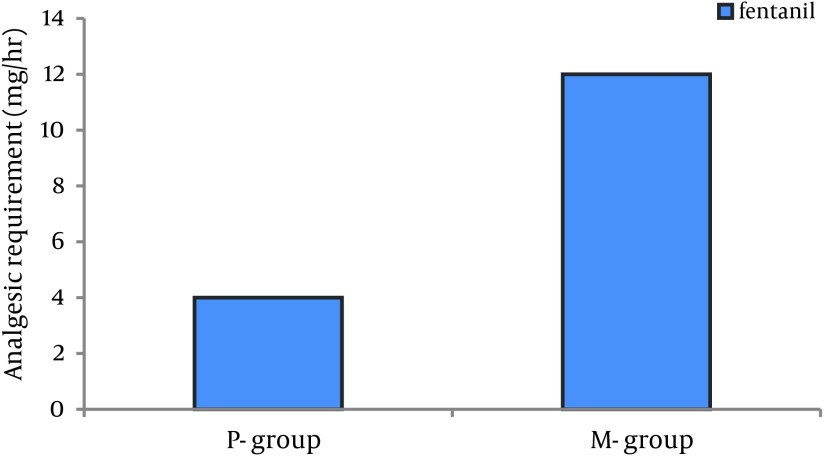
Fentanyl Requirements for Patients Receiving Midazolam and Propofol While Mechanically Ventilated in the ICU M-group, Midazolam group; P-group, Propofol group.

**Figure 3.**
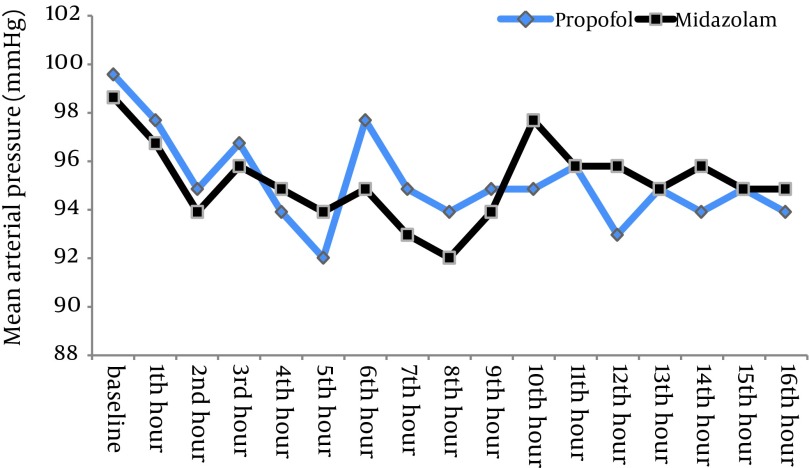
Mean Arterial Pressure in Groups on ICU Arrival, During Sedative Infusion and on Sedative Discontinuation

**Figure 4.**
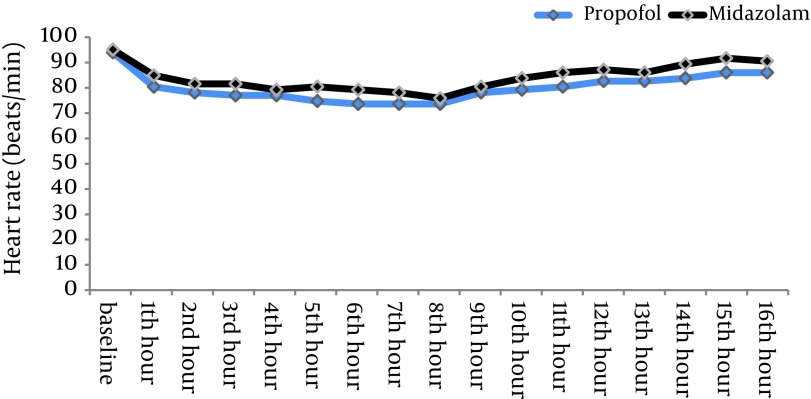
Mean Heart Rate in Groups on ICU Arrival, During Sedative Infusion and on Sedative Discontinuation

**Figure 5.**
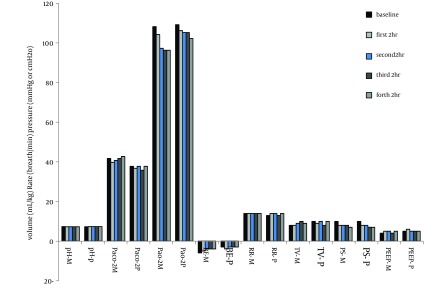
Mechanical Ventilation Variables and Arterial Blood Gas Analysis in the First eight Hours of Intubation and Mechanical Ventilation

There were no statistically significant differences between the two patient groups with respect to age, weight, gender and left ventricular ejection fraction (Table 1). Mean duration of sedative infusion in ICU was 10 hours (8-12 hr) in the propofol group and 12 hours (9-15 hr) in the midazolam group (P > 0.05). Only one patient in the propofol group had criteria for extubation before 8 hours and so received sedation less than 8 hours. There were no significant differences in mean Ramsay sedation scores between two groups during assisted ventilation (4.5 for the midazolam group vs 4.7 for the propofol group; P = 0.259). There was no difference in time percentage within the target RSS range between two groups (77.3% for propofol group vs 75.1% for midazolam group; P = 0.18). Patients receiving midazolam infusions required significantly more fentanyl [mean = 12.5 (11–14.5) mg/hr] than patients receiving propofol [mean = 4 (3.25–6) mg/hr] (P = 0.0039) (Figure 2). The midazolam group received about three times more fentanyl compared with patients sedated with propofol. Sixteen percent of patients in the midazolam group (n = 4) and 28% of patients in propofol group (n = 7) did not require an analgesic drug (P = 0.49). No significant differences were seen between the baseline and throughout the study values of BPs and HRs in two groups (Figures 3 , 4). There were no differences in heart rate (P = 0.41) and mean arterial pressure between the two groups (P = 0.51) during sedative infusion and at the time of sedative discontinuation. Mechanical ventilation variables and arterial blood gas analysis were similar between the two groups for the first 8 h of intubation and artificial ventilation. There were no significant differences in arterial blood gas parameters and mechanical ventilation variables between the two groups at the baseline and throughout the study (P > 0.05) (Figure 5). Extubation times in patients sedated with propofol were shorter than those sedated with midazolam (102 ± 27min vs 245 ± 42 min, respectively; P < 0.05) following discontinuation of the sedation but there was no significant difference in ICU discharge time (47.5 hr vs 36.3 hr, respectively) between the two groups (P = 0.24). There were no respiratory adverse events after extubation in both groups, and no patient required re‐intubation.

**Table 1.** Demographic and Cardiac Variable Status

	P-Group ^a^	M-Group ^a^	P value
**Age, Mean ± SD, y**	51 ± 4.2	52 ± 4.5	0.52
**Weight, Mean ± SD, kg**	73 ± 19	69 ± 17	0.82
**Gender, Male, No. (%)**	19 (76)	14 (56)	0.23
**LVEF ^a^, Mean ± SD**	45 ± 4.2	45 ± 4.0	0.75

^a^ Abbreviations: LVEF, left ventricular ejection fraction; M-Group, midazolam group; P-Group, propofol group.

## 5. Discussion

Until recently, ICU care focused on correcting medical/surgical issues without worrying about the oversedation or prolonged ventilation time. This randomized trial study was designed to compare the propofol based sedation for post-CABG patients with the midazolam-based sedation regimens in the ICU. In our study, an equivalent depth of sedation between midazolam and propofol receiving ICU patients was achieved. These results are consistent with previous studies in patients admitted to ICU after a variety of major surgeries (15-17, 25-27). On the other hand, studies have shown that the practice of keeping patients heavily sedated during mechanical ventilation extends their stay time in the ICU, but the use of short acting sedative drugs like propofol can solve this problem. This did not happen in our study for the propofol receiving patients. Because propofol alone has no analgesic activity, opioids are given to control pain (20), but not in all patients (18). The propofol-sedated patients in this study required significantly less analgesia and thus respiratory stability was not compromised. Propofol may cause hypotension specially in patients who have limited myocardial reserve (28) and also respiratory depression, which can be exaggerated in the presence of opioids (29). As a result, we have adapted its use to minimize these risks and avoid respiratory depression by using minimal dose of propofol that is suitable for maintaining target level of sedation and discontinuing sedative and analgesic drugs before weaning patients off the ventilator. Some other clinicians like Kress et al. (30) improved patient outcomes with a daily administration of the sedatives. Prolonged tracheal intubation and mechanical ventilation may be associated with adverse clinical events, including development of nosocomial pneumonia (31) and barotrauma (32). So drugs that reduce the time that a patient receives mechanical ventilation should lead to reduction in such adverse events. There are many studies which have shown propofol is more effective compared with midazolam regarding the quality of sedation, and shortening of the time between the termination of sedation and extubation (18, 19), but not necessarily the ICU stay time. Our trial confirms the findings of the majority of previous randomized studies, which have demonstrated more rapid times for awakening (15-17, 27, 33, 34) and reduced times for tracheal extubation (18, 19) with the use of propofol for ICU sedation but not regarding the ICU stay time (33, 34). However, Higgins et al. did not find a difference in time for tracheal extubation when comparing propofol to midazolam for sedation in a cardiac surgical patient population.The hemodynamics of propofol has been shown in the previous studies, in patients under anaesthesia, (26, 27) and, more recently, ICU patients (35, 36). Vasodilatation, which manifests itself as a reduction in arterial pressure, is a feature of sedation with both propofol (27, 37) and midazolam (38). In this study, equipotent sedative doses of these agents, infused in patients, resulted in equivalent mild reductions in arterial pressures and heart rates. Our results about the length of the ICU stay might not be valid due the small sample size. In summary, although propofol is safe and effective for the postsurgical sedation of CABG patients when compared with midazolam-based sedation, further studies with larger sample sizes are needed to have a firm conclusion regarding all effects of this sedative drug.

### 5.1. Study limitations

Inadequate sample size to evaluate some issues such as the length of ICU stay.

Sedation and analgesia are essential components of care for many mechanically ventilated patients in the intensive care unit (ICU). This study and several other studies during recent years have shown propofol to be an effective and safe agent for use as post‐operative sedation in the ICU. Propofol and midazolam are commonly used for the care of post–coronary artery bypass graft (CABG) patients in the ICU, but compared with midazolam, propofol provides equal or better control in maintaining the sedation and more rapid recovery, more rapid extubation when sedation is terminated and less requirement for analgesic drugs for pain control. These properties have advantages for patients at risk for myocardial ischemia.
